# Isoform-Specific Upregulation of Palladin in Human and Murine Pancreas Tumors

**DOI:** 10.1371/journal.pone.0010347

**Published:** 2010-04-26

**Authors:** Silvia M. Goicoechea, Brian Bednarski, Christianna Stack, David W. Cowan, Keith Volmar, Leigh Thorne, Edna Cukierman, Anil K. Rustgi, Teresa Brentnall, Rosa F. Hwang, Christopher A. G. McCulloch, Jen Jen Yeh, David J. Bentrem, Steven N. Hochwald, Sunil R. Hingorani, Hong Jin Kim, Carol A. Otey

**Affiliations:** 1 Department of Cell and Molecular Physiology, University of North Carolina, Chapel Hill, North Carolina, United States of America; 2 Division of Surgical Oncology, Department of Surgery, University of North Carolina, Chapel Hill, North Carolina, United States of America; 3 Lineberger Comprehensive Cancer Center, Chapel Hill, North Carolina, United States of America; 4 Department of Pathology and Laboratory Medicine, University of North Carolina, Chapel Hill, North Carolina, United States of America; 5 Cancer Biology Program, Fox Chase Cancer Center, Philadelphia, Pennsylvania, United States of America; 6 Departments of Medicine and Genetics, Abramson Cancer Center, University of Pennsylvania, Philadelphia, Pennsylvania, United States of America; 7 Department of Medicine, University of Washington, Seattle, Washington, United States of America; 8 Department of Surgical Oncology, M. D. Anderson Cancer Center, University of Texas, Houston, Texas, United States of America; 9 CIHR Group in Matrix Dynamics, University of Toronto, Toronto, Canada; 10 Surgical Oncology, Northwestern Medical Faculty Foundation, Chicago, Illinois, United States of America; 11 Division of Surgical Oncology, University of Florida, Gainesville, Florida, United States of America; 12 Fred Hutchinson Cancer Research Center and University of Washington, Seattle, Washington, United States of America; Virginia Tech, United States of America

## Abstract

Pancreatic ductal adenocarcinoma (PDA) is a lethal disease with a characteristic pattern of early metastasis, which is driving a search for biomarkers that can be used to detect the cancer at an early stage. Recently, the actin-associated protein palladin was identified as a candidate biomarker when it was shown that palladin is mutated in a rare inherited form of PDA, and overexpressed in many sporadic pancreas tumors and premalignant precursors. In this study, we analyzed the expression of palladin isoforms in murine and human PDA and explored palladin's potential use in diagnosing PDA. We performed immunohistochemistry and immunoblot analyses on patient samples and tumor-derived cells using an isoform-selective monoclonal antibody and a pan-palladin polyclonal antibody. Immunoblot and real-time quantitative reverse transcription-PCR were used to quantify palladin mRNA levels in human samples. We show that there are two major palladin isoforms expressed in pancreas: 65 and 85–90 kDa. The 65 kDa isoform is expressed in both normal and neoplastic ductal epithelial cells. The 85–90 kDa palladin isoform is highly overexpressed in tumor-associated fibroblasts (TAFs) in both primary and metastatic tumors compared to normal pancreas, in samples obtained from either human patients or genetically engineered mice. In tumor-derived cultured cells, expression of palladin isoforms follows cell-type specific patterns, with the 85–90 kDa isoform in TAFs, and the 65 kDa isoform predominating in normal and neoplastic epithelial cells. These results suggest that upregulation of 85–90 kDa palladin isoform may play a role in the establishment of the TAF phenotype, and thus in the formation of a desmoplastic tumor microenvironment. Thus, palladin may have a potential use in the early diagnosis of PDA and may have much broader significance in understanding metastatic behavior.

## Introduction

Pancreatic adenocarcinoma is the fourth leading cause of cancer death in the United States [1]. This disease has an exceptionally high lethality rate, due to its aggressive metastasis and the low probability of diagnosis at an early stage. Approximately 80–90% of patients with pancreatic cancer present with locally-advanced, unresectable tumors or metastatic disease at the time of initial diagnosis [2], [3]. The dismal prognosis associated with pancreatic adenocarcinoma has driven a search to identify the aberrant signaling pathways that contribute to the development, growth, and invasion of this disease, with the ultimate goal of developing novel diagnostic biomarkers and effective targeted therapies [4].

Palladin is a cytoskeleton-associated scaffold protein that has received attention recently in the pancreas cancer field [5], [6]. Palladin's function in normal cells has been defined previously by knockdown and overexpression experiments in cultured cell models, and it is clear that palladin is critically involved in actin-dependent behaviors such as cell motility and contractility [7], [8], [9], [10]. In animal studies, palladin is upregulated during wound-healing [11], [12], [13], and it is required for normal mammalian embryogenesis [14]. In human breast cancer cells, high levels of palladin expression are associated with increased invasiveness [15], [16], which suggests the possibility that abnormalities in palladin expression or function might contribute to the disregulated motility of metastatic cancer cells. Palladin's precise role in pancreas cancer has not yet been defined; however, a mutation in the human palladin gene is associated with a rare form of familial pancreatic cancer. Palladin was found to be overexpressed in samples of sporadic pancreatic adenocarcinoma and in tumor-derived cell lines [5]. These results were challenged by a subsequent study that utilized immunohistochemical (IHC) staining of a pancreas tumor array [6]. Although the follow-up study confirmed that palladin is overexpressed in 96% of pancreas tumors as compared to normal pancreas, it showed that palladin is upregulated in stromal fibroblasts rather than in the neoplastic cells of pancreas tumors [6]. Thus, the results provide evidence that palladin is overexpressed specifically in pancreas tumors, yet the identity of the cell type that is responsible for upregulating palladin in these tumors remains unclear.

Palladin exists in all vertebrates as multiple size variants generated from a single gene that possesses alternative promoters, i.e. a “nested gene”. There are three major palladin isoforms that arise from alternative start sites (85–90, 140 and 200 kDa) and multiple minor isoforms that result from alternative splicing [10], [17], [18], [19]. This rich diversity of isoforms raises the possibility that human cells may express palladin variants that are not detected by all antibodies, which could be the cause of previous conflicting results. In the current study, w e set out to perform Western blot analysis and immunohistochemical staining of pancreatic tumors and tumor-derived cells using both isoform-selective monoclonal and pan-palladin polyclonal antibodies, to identify all of the palladin isoforms that are associated with the different cell types found in pancreatic tumors. We show that there are two major isoforms of palladin in pancreatic tumors: 65 kDa and 85–90 kDa. PDA expresses predominantly the 85–90 kDa isoform of palladin, while normal pancreas and non-PDA tumors both express the 65 kDa isoform. We also show that palladin overexpression occurs primarily in tumor-associated fibroblasts (TAFs), and not the neoplastic epithelial cells, of human pancreatic tumors. These results suggest the possibility that upregulation of 85–90 kDa palladin may be a critical step in the acquisition of the activated fibroblast phenotype, which is key to the formation of a pro-invasive tumor microenvironment.

## Results

### Human Palladin Isoforms are Diverse

**Figure 1:**
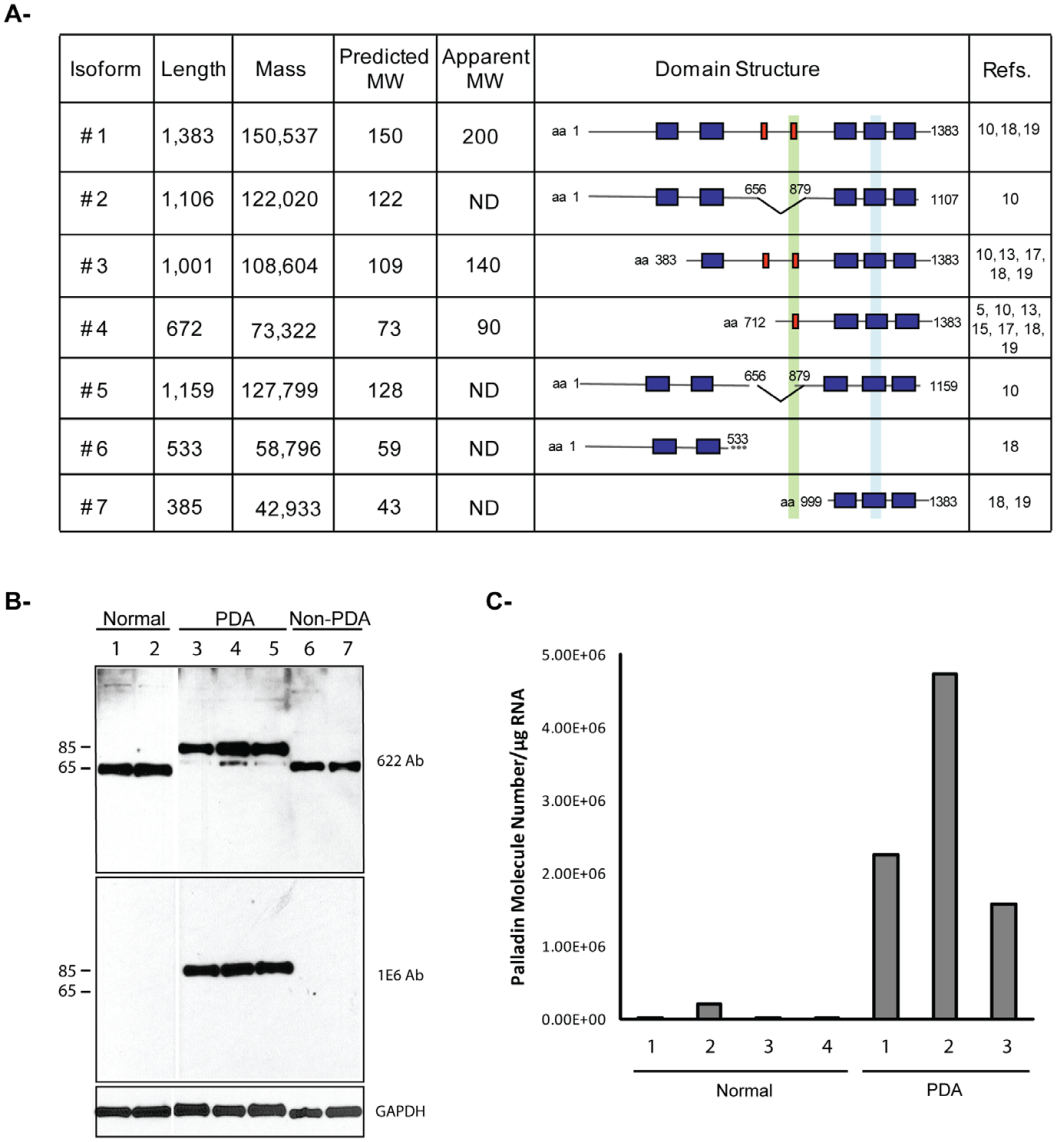
Analysis of human palladin isoforms in pancreatic tissues. **A.** **Human palladin isoforms.** Proline-rich domains are represented by red boxes, and Ig-like domains are shown as blue boxes. The epitope recognized by the 1E6 and 4D10 antibodies is highlighted in green. The region amplified by RT-qPCR is highlighted in light blue. Isoform #1, 3 and 4 are the primary products of the palladin gene and have been detected by immunoblotting. The sequences of these isoforms are published. The sequences of isoforms #2, 5, 6, and 7 were obtained from genomic databases. “ND”: not-determined. **B.** **Western blot analysis of pancreas samples.** Small pieces of fresh tissue were snap-frozen in liquid nitrogen, ground in a chilled mortar and pestle, extracted in a detergent-containing lysis buffer, and centrifuged at 15,000×g to remove any unsolubilized particulates. The supernatant was boiled in Laemmli sample buffer and resolved by SDS-PAGE, with 15 µg protein loaded per lane. The samples were immunoblotted and probed with two anti-palladin antibodies and an antibody to GADPH (a housekeeping gene) as a control for equal loading. Lanes 1–2: normal pancreas. Lanes 3–5: primary adenocarcinoma tumors (PDA). Lane 6–7: Non-primary adenocarcinoma tumors (Non-PDA) (Lane 6: solid pseudopapillary tumor, Lane 7: neuroendocrine tumor). **C.** **RT-qPCR.** Total RNA was isolated from normal tissue (patients 1–4) and PDA tumors (patients 1–3), reverse transcribed, and subjected to RT-qPCR using gene-specific primers. Each bar represents the mean + SEM (0.06–0.35%) from three or more independent determinations.

Palladin is a highly conserved protein that is found in all vertebrate species, and it arises from a single gene in mice and humans (*PALLD*). The palladin gene is unusually large and complex, spanning ∼400 kb and containing at least 25 exons, and it produces multiple palladin isoforms, including three well-described size variants that resolve at 85–90, 140 and 200 kDa [10], [17], [18], [19]. In addition to the three classical palladin isoforms, size variants resolving at ∼115, 75, 65 and 50 kDa have been reported in previous studies [6], [10], [14], [19], suggesting that palladin isoforms are more diverse than previously suspected. This motivated us to carefully assess the existing databases of full-length transcripts to look for evidence of additional palladin isoforms. Examination of the Universal Protein database revealed the existence of seven palladin variants in humans (http://beta.uniprot.org/uniprot/Q8WX93), and alignment of their sequences is shown in Figure 1A. It has been noted previously that some palladin isoforms display a higher molecular weight by SDS-PAGE (apparent MW) than would be predicted based upon their sequence, and this is believed to be a consequence of their high proline content, and this is reflected in Figure 1A. Palladin contains two different conserved domains that contribute to its role as an actin-binding scaffolding molecule: proline-rich domains that are hot-spots for protein-protein interaction, and Ig domains that mediate F-actin crosslinking [15]. Since the various palladin isoforms each possess different combinations of proline-rich and Ig domains, it is likely that they serve distinct cellular functions. Therefore, we went on to determine which palladin isoforms are expressed in pancreas, and if these palladin isoforms follow cell-type specific patterns of expression in pancreatic cells.

**Figure 2:**
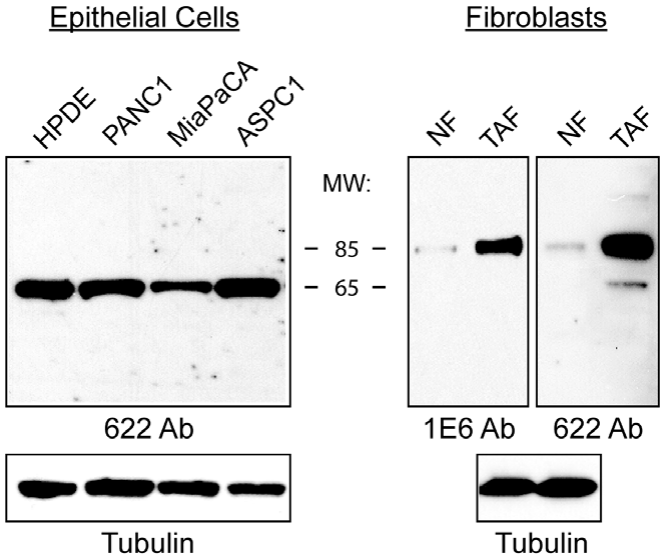
Palladin expression in pancreatic cancer cells. Immunoblot analysis of pancreatic tumor-derived cell lines, tumor-associated fibroblasts, and corresponding normal cells. Pancreatic cells: normal human pancreatic ductal epithelial cells (HPDE) and three tumor cell lines: PANC1, MiaPaCA, and ASPC1. Fibroblasts: normal adult fibroblasts (NF) and tumor-associated fibroblasts (TAF). Whole cell lysates were analyzed by western blot using the polyclonal antibody 622. Blots were also stained for tubulin as a control for equal loading.

Two previous studies have explored palladin expression in cultured cells derived from pancreas tumors, and both focused on the widely-expressed 85–90 kDa palladin isoform [5], [6], yet these previous studies also presented evidence that pancreatic tumor-derived cell types express smaller, uncharacterized palladin variants (see, for example, the 75 kDa band in Fig. 2 of reference 6). Neither previous study investigated palladin expression in bulk tumor samples, which contain a complex mixture of different cell types. Therefore, we used Western blot analysis of donated patient samples to determine which palladin isoforms are upregulated in human tumors, and if palladin levels are associated with the invasiveness of the tumors. For these experiments, we analyzed samples of normal pancreas (Fig. 1B, lanes 1, 2) and primary pancreatic ductal adenocarcinomas (PDA, lanes 3–5), and also two different types of non-PDA tumors: a solid pseudopapillary tumor of the pancreas (a tumor characterized as having lower metastatic potential and favorable prognosis even with large primary tumors) and a neuroendocrine carcinoma (which was diagnosed and resected at an early, non-invasive stage). Lysates of these tumors are shown in lanes 6 and 7, respectively, in Figure 1B. A polyclonal palladin antibody designated 622 and an isoform-selective monoclonal antibody called 1E6 (see Figure 1A) were used to stain parallel immunoblots. Immunodetection with the 622 polyclonal revealed that normal pancreas and non-PDA tumors both express an isoform resolving at ∼65 kDa, while PDAs express predominantly a palladin band resolving near 85–90 kDa and a trace amount of the 65 kDa isoform. When parallel immunoblots were stained with the 1E6 monoclonal, only the 85–90 kDa band was detected (140 and 200 kDa palladin isoforms are not expressed in pancreas). This is consistent with previous studies, in which the 1E6 monoclonal was found to be selective for the 85–90 kDa isoform of palladin [10], [18]. It is important to note that the upregulation of 85–90 kDa palladin shown in Figure 1B is associated specifically with PDAs, which are both highly invasive and strongly desmoplastic, and not with tumor types that are only moderately invasive and associated with a less robust expansion of the stroma. To confirm these results, we performed RT-qPCR in an independent set of samples. The primers used for this study should, in theory, recognize all palladin isoforms that contain the Ig4 domain (i.e., all except isoform #6). Consistent with the immunoblots results, Figure 1C shows that the levels of palladin mRNA in PDA samples are highly elevated compared with normal pancreas. This demonstrates that western blot and RT-qPCR are feasible methods for quantifying the 85–90 kDa isoform of palladin (protein and mRNA) and thus, may have a potential use in the diagnosis of pancreatic ductal adenocarcinoma.

### 85–90 kDa Palladin Isoform is Strongly Upregulated in Tumor-associated Fibroblasts

To resolve the existing confusion about the level of palladin expression in different types of tumor-derived cells, we used both the broadly-reactive polyclonal antibody 622 and the isoform-selective monoclonal 1E6 to compare the pattern of isoforms detected in tumor-associated fibroblasts and neoplastic tumor cell lines. For comparison, we also analyzed the corresponding non-tumor cells types. In Figure 2, human pancreatic ductal epithelial (HPDE) cells were used as normal controls and probed in parallel with three different lines of PDA-derived cells (Panc-1, MiaPaCa, AsPC-1). The polyclonal antibody 622 detected a single 65 kDa isoform in HPDE cells and all three tumor cell lines, and the expression level of this palladin isoform did not appear to vary significantly between normal and tumor cells. When parallel blots were probed with monoclonal 1E6, no bands were detected, even at long exposure times (Figure S1). In contrast, when normal human fibroblasts (labeled NF) were analyzed alongside lysates of primary pancreatic tumor-associated fibroblasts (TAF), the results showed that 85–90 kDa palladin was strongly up-regulated in TAFs, and this band was detected with both antibodies (1E6 and 622). In addition, the polyclonal 622 antibody detected a minor band at 65 kDa in the TAFs. To confirm these results, we also analyzed a newly available line of immortalized pancreatic tumor-derived stellate cells (PSC), which closely resemble TAFs in their phenotype and patterns of gene expression [20], and the results obtained with these cells were identical to those obtained with primary TAFs (data not shown). These results suggest that cells of epithelial origin may express different palladin isoforms than cells of mesenchymal origin, and also confirm that a 85–90 kDa palladin isoform is robustly upregulated in tumor-associated fibroblasts when compared to normal adult fibroblasts, as reported previously [6].

**Figure 3:**
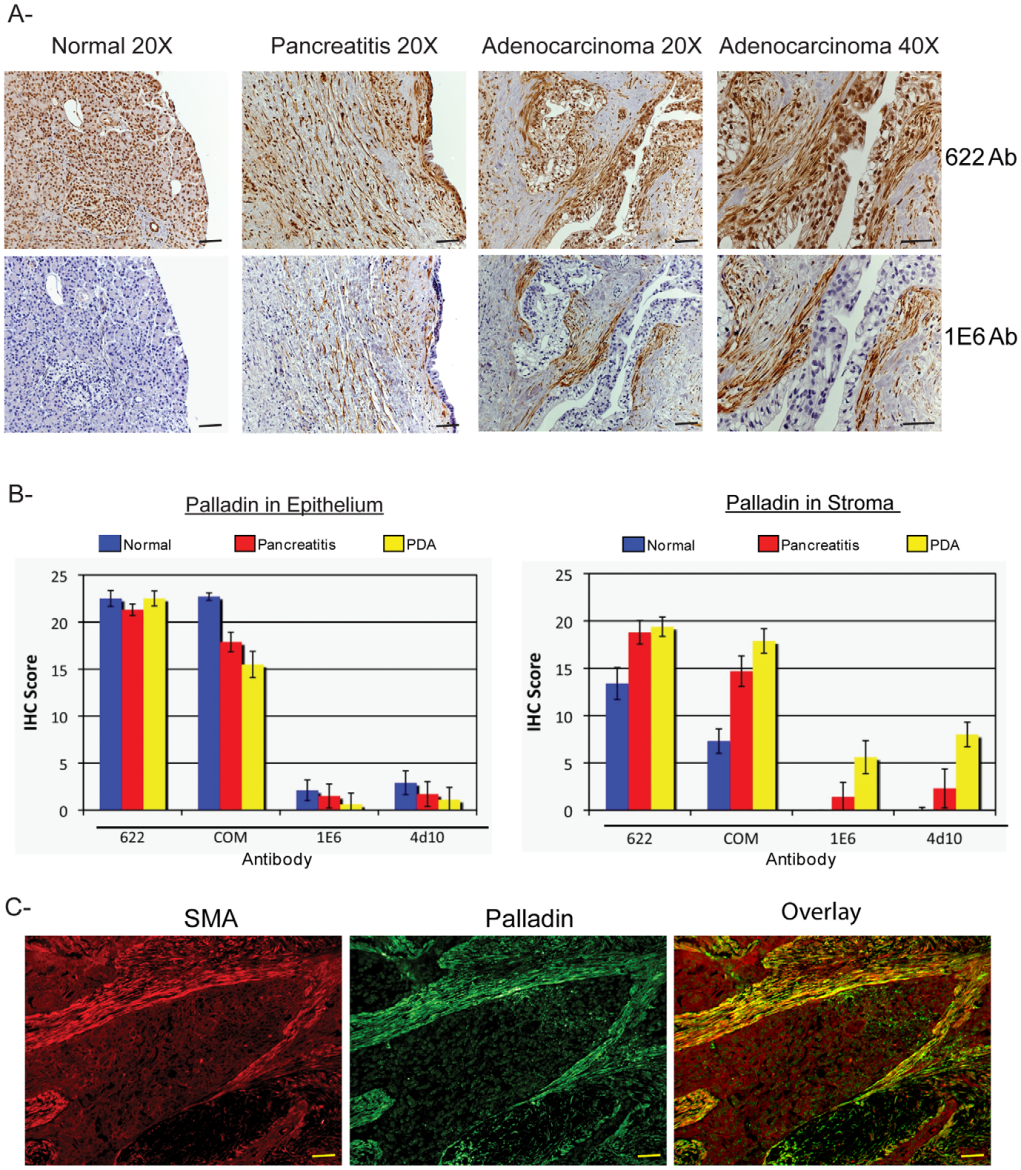
Palladin staining of paraffin-embedded patient tissues. **A.** IHC staining was performed using standard antigen-retrieval protocols, and counter-stained with hematoxylin. Tissue sections were stained for palladin using two palladin antibodies: polyclonal 622 and monoclonal 1E6. Palladin stain is detected with brown reaction product. In tumor sections, palladin is detected at dramatically elevated levels in the stromal fibroblasts. Note also the expanded stroma around the neoplastic cells, which is characteristic of the desmoplastic reaction. Scale bars, 200 µM. **B.** **Quantification of immunohistochemistry results.** Ten sections each of normal pancreatitis and adenocarcinoma specimens were stained with four different antibodies (622, COM, 1E6 and 4D10) and scored by two pathologists, as described in the text. Results for both ductal epithelium (left) and stroma (right) stained with various palladin antibodies are shown for normal pancreas (n = 9, blue), pancreatitis (n = 7, red) and pancreatic adenocarcinoma (n = 10, yellow). The results confirmed that palladin levels are increased in the stroma, and not the epithelial tumor cells, of the adenocarcinomas. Although palladin levels are also increased in cases of chronic pancreatitis, they do not reach the same levels as in the tumors. Compared to the polyclonal 622 and COM, the monoclonal antibodies 1E6 and 4D10 are effective at distinguishing between pancreatitis and cancer. **C.** Double-label immunostaining for palladin (1E6 Ab) and α-SMA in sections of pancreatic tumors confirms that palladin is strongly detected in a population of activated TAFs that surround the neoplastic cells. Scale bars, 200 µM.

To provide insight into the cellular distribution of palladin within pancreatic tumors, we used polyclonal and monoclonal palladin antibodies for IHC staining of paraffin-embedded patient specimens, including normal pancreas, pancreatitis, and pancreatic adenocarcinoma. Multiple sections of each type were stained with polyclonal and monoclonal palladin antibodies. As expected from the cultured cell immunoblots, we found that the polyclonal antibody 622 generated moderately intense staining in section of normal pancreas. Immunostaining was detected with this antibody in the cytoplasm and also the nuclei of normal pancreatic cells (Fig. 3A, top row). In samples of pancreatitis, which is a benign fibrotic disease characterized by the presence of activated fibroblasts, both nuclear and cytoplasmic staining was also observed in stromal fibroblasts with polyclonal antibody 622. In samples of pancreatic adenocarcinoma, particularly intense staining was seen in the stroma, but also more faintly in epithelial cells with polyclonal 622. By contrast, when normal pancreas was stained with palladin monoclonal 1E6, only a faint blush of staining was observed (Fig. 3A, bottom row), which is consistent with the observation that this antibody detects an isoform that is not expressed at detectable levels in normal cells of epithelial origin. In samples of pancreatitis, moderate staining of stromal fibroblasts was detected with monoclonal 1E6, and intense staining of the stroma was detected in sections of pancreatic ductal adenocarcinoma (Fig. 3A, bottom row). Taken together with the immunoblot results shown in Fig. 2, the IHC staining patterns obtained with monoclonal antibody 1E6 suggests that a 85–90 kDa palladin isoform is dramatically upregulated in the activated stromal fibroblasts of pancreatic adenocarcinoma tumors, and to a lesser degree in the stroma associated with fibrotic disease.

To provide additional confirmation of these results, a second polyclonal antibody from a commercial source (designated COM, and obtained from ProteinTech Group), and a second isoform-selective monoclonal antibody (4D10) were used to stain multiple sections of pancreas (normal, pancreatitis, and PDA). It should be noted that the COM antibody was used in a previous study to for IHC staining of pancreas tumors (6). The results obtained with the COM antibody were very similar to those generated with the polyclonal 622, and the staining pattern obtained with 4D10 was virtually indistinguishable from that obtained with monoclonal 1E6 (Figure S2). Negative control staining for IHC was performed in paraffin-embedded human pancreatic tissues (Figure S3) and as expected, no signal was detected without application of primary antibody. To quantify these results, the slides were reviewed and scored by two independent pathologists blinded to the tissue diagnoses and to the specific antibodies uses. Each specimen was assigned a score for staining intensity (values 1–4, with 4 representing the most intense stain) and a score for percentage of positively stained cells (values 0–6, with 6 representing greater than 75%). The values for intensity of staining and percentage of positive cells were then multiplied to yield a combined score for each sample (range from 0 to 24). Each tissue sample was scored twice: once for the presence of positive staining in the ductal epithelium and once for the presence of positive staining in the stroma. Figures 3B display these findings graphically as average score plotted as a function of tissue type. Results for both ductal epithelium and stroma stained with various palladin antibodies are shown for normal pancreas (n = 9, blue), chronic pancreatitis (n = 7, red) and pancreatic adenocarcinoma (n = 10, yellow). This quantification confirms that palladin expression is dramatically higher in the stroma, but not the ductal epithelial cells, of pancreas tumors as compared to either normal pancreas or pancreatitis.

Our IHC results are in agreement with an earlier study, which reported that palladin is strongly overexpressed in the stroma of 171 of 177 sections (96.6%) of PDA tumors [6]. In that study, palladin-positive stromal cells were identified as tumor-associated fibroblasts based upon their morphology. To definitively confirm the identity of palladin-positive cells within the pancreatic tumors, we performed double-label immunofluorescence staining on tumor sections, using the palladin monoclonal 1E6 and a monoclonal antibody to α-smooth muscle actin (α-SMA), a marker for fibroblast activation. As shown in Figure 3C, in sections of pancreatic adenocarcinoma, the staining patterns for palladin and α-SMA co-localized, which supports the conclusion that palladin is expressed in stromal cells expressing α-SMA, which include activated TAFs and stellate cells.

### Palladin is Upregulated in Lymph Node and Liver Metastases and in Other Human Cancers

**Figure 4:**
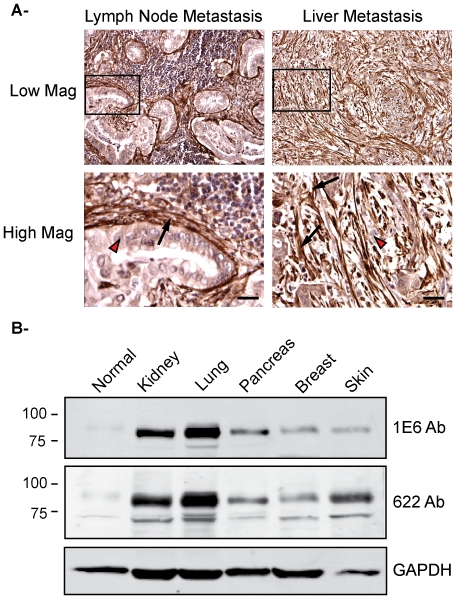
Palladin in human pancreatic cancer metastasis and other human cancers. **A.** **Immunohistochemistry of lymph node and liver metastases.** Formalin-fixed, paraffin-embedded tissue sections were stained with COM antibody. Left: Low and high magnification images of lymph node metastasis. Right: Low and high magnification of liver metastasis. Arrow heads, lightly-stained tumor epithelial cells. Arrows, intensely-stained tumor-associated fibroblasts. Scale bars, 100 µM. **B.** **Immunoblot analysis of tumor-associated fibroblasts from different human cancers.** Tumor-associated fibroblasts were isolated from different human cancers: normal, kidney, lung, pancreas, breast and skin. Whole cell lysates were analyzed by western blot using the monoclonal antibody 1E6 and the polyclonal antibody 622. Blots were also stained for GAPDH as a control for equal loading.

Two previous reports have suggested a potential role for palladin in the metastasis of breast cancer by showing that palladin levels are elevated in a highly invasive subpopulation of human breast cancer cells [15], [16]. The propensity for early invasion and metastasis is a characteristic trait of pancreas tumors, such that we were prompted to explore palladin expression in samples of pancreatic cancer metastases to lymph nodes and liver to determine if palladin is upregulated in the tumor cells or the TAFs in these samples. As demonstrated in Figure 4A, palladin staining was strongly detected in TAFs, and only weakly in the neoplastic epithelial cells, in both types of metastases. These results suggest that neoplastic cells are able to induce palladin upregulation in neighboring stromal cells as a mechanism to recreate a favorable microenvironment following metastatic invasion to a new location.

The results shown so far indicate that the 85–90 kDa palladin isoform is upregulated in *PDA* tumor-associated fibroblasts in both primary and metastatic tumors. To extend our observations to other human cancers we performed immunoblot analysis of tumor-associated fibroblasts isolated from five different tumors obtained from breast, lung, ovary, kidney and skin. Pancreas was included as a control. We used both the polyclonal antibody 622 and the monoclonal 1E6 to compare the pattern of isoforms detected in tumor-associated fibroblasts. As expected, Figure 4B shows that the 85–90 kDa palladin isoform is upregulated tumor-associated fibroblasts from all tumors analyzed indicating that 85–90 kDa palladin upregulation in the stromal microenvironment may be a common feature of human cancers.

### Palladin Expression in Murine PDA Recapitulates the Pattern Seen in Human PDA

**Figure 5:**
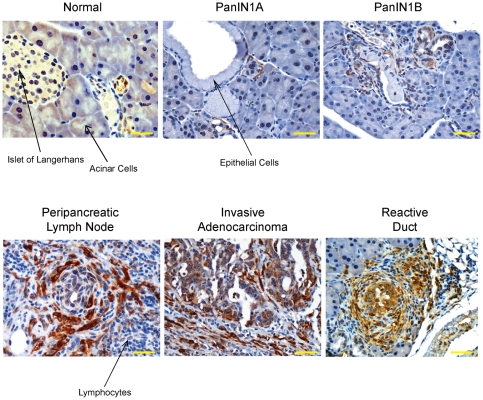
Palladin expression in LSL-KrasG12D/+ mice. Normal, primary tumors and metastasis tissue sections were stained with 622 Ab. Palladin staining is noted in stromal fibroblasts in both primary adenocarcinomas and metastases. Scale bars, 50 µM.

Important insights into the mechanisms of pancreatic adenocarcinoma pathogenesis have been gained from the use of genetically engineered mice that express an activating mutation in the Kras gene (*Kras^G12D^*) targeted to the pancreas [21], [24]. We performed immunohistochemical analyses on primary tumors and metastases harvested from these mice to characterize palladin expression during disease progression. Beginning with the earliest precursor ductal lesions (i.e. PanIN-1A and -1B), associated fibroblasts show staining for palladin using either antibody 622 (Figure 5) or the commercially available polyclonal antibody (data not shown). Control staining for IHC of paraffin-embedded mouse pancreatic tissue is shown in Figure S3. Interestingly, palladin expression in the reactive fibrosis associated with acinar-ductal metaplasia regions (around the so-called “reactive ducts”) was even more robust (Figure 5). We note that the ductal epithelial cells in the adjacent PanINs do not show palladin expression, nor do the surrounding normal acinar parenchymal cells. Palladin-expressing fibroblasts increase in number and staining intensity with disease progression, as strongly positive TAFs were seen diffusely infiltrating the invasive ductal adenocarcinomas, along with occasional faint cytoplasmic staining of the primary tumor cells (Figure 5). Interestingly, in other areas of invasive disease with scant to non-existent stromal cells, the tumor cells are completely negative. The specificity of clustering of palladin-positive TAFs around tumor cells is clearly demonstrated in peripancreatic lymph nodes with infiltrating disease (Figure 5). The TAFs form distinct rings around nests of tumor cells while the intervening regions containing lymphocytes are completely negative. Taken together with the results from human PDAs, these findings suggest that upregulation of palladin by TAFs is likely to be a common feature of desmoplastic tumors in multiple species.

### Palladin Isoforms in Needle-Core Biopsy Samples

**Figure 6:**
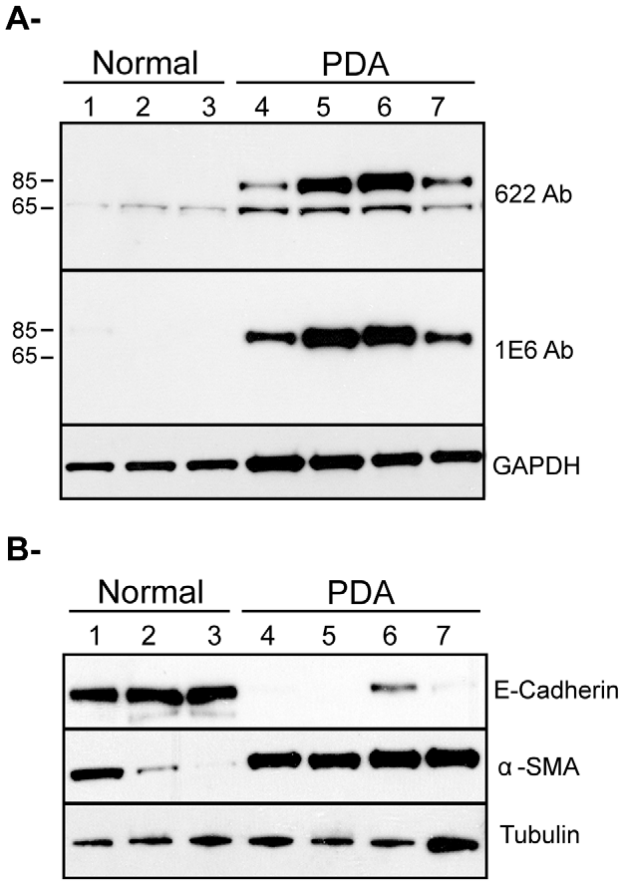
Detection of palladin in post-surgical samples collected with 18-gauge needles. **A.** Samples of normal (lane 1 to 3) and pancreatic adenocarcinoma (lanes 4 to 7) were obtained from donated post-surgical organs using 18-gauge needles. Tissue samples were snap-frozen, ground, lysed and analyzed as in Figure 1. The blot was stained with both monoclonal (1E6) and polyclonal (622) palladin antibodies, and the major band (85–90 kDa) was detected by both antibodies in all tumor samples. **B.** Same samples (normal, lane 1–3 and PDA, lane 4–7) were analyzed for epithelial vs myofibroblast markers. The blot was stained with both, anti-E-cadherin antibody (as an epithelial cell marker) and anti-αSMA antibody (as a myofibroblast marker). Blots were stained for tubulin as a control for equal loading.

The results of both IHC staining and immunoblot analysis suggest that immunodetection of palladin by isoform-selective monoclonal antibodies might have utility in diagnosing pancreatic adenocarcinoma. However, it should be acknowledged that the amount of tissue used for immunoblot analysis in Fig. 1B is greater than the amount that is typically collected by clinicians for patient diagnosis, as the starting material that was homogenized and lysed in that analysis consisted of pea-sized fragments, ∼30–50 mgs each, while the material available to a diagnostician normally consists of 10–15 mg if collected by needle-core biopsy. Thus, to determine if differences in palladin expression could be detected using smaller samples, we also analyzed specimens collected from donated post-surgical organs using 18-gauge needles. In Figure 6A, lane 1–3 contains samples of normal pancreas, and lanes 4–7 contain samples from the adenocarcinoma tumors of four different patients. We used monoclonal antibody 1E6 and polyclonal 622 to stain lysates of the needle core samples. As shown in Figure 6, only the major palladin isoform (85–90 kDa) is detected in all the tumor samples, and palladin levels were strikingly elevated in all tumor samples when compared to the control samples. The results presented so far in this study indicate that upregulation of 85–90 kDa palladin occurs in the stromal fibroblasts (Figure 2 and 3). To extend our observations to samples collected by needle-core biopsy, we performed immunoblot analysis to determine the origin of cells within the patient samples that express high levels of 85–90 kDa palladin. We used E-cadherin antibody as an epithelial marker and an α-SMA antibody as a myofibroblast marker (Figure 6B). Figure 6A and 6B demonstrate that normal pancreas samples contain abundant E-cadherin expressing epithelial cells, as expected, while tumor samples contain a high proportion of α-SMA-expressing cells, which presumably represent the palladin expressing tumor-associated fibroblasts. It should be noted that these samples contain a high degree of cellular heterogeneity, such that the detection of moderate levels of α-SMA in one normal sample (lane 1) may represent the presence of contaminating vascular smooth muscle cells within that biopsy, and the detection of a small amount of E-cadherin in one tumor sample (lane 6) probably reflects the presence of a small amount of normal pancreas within that sample. Nevertheless, the overall pattern of these immunoblot results support the conclusion that 85–90 kDa palladin is upregulated in TAF-containing tumor samples, when compared to normal pancreas samples.

## Discussion

We show by multiple techniques using patient samples, cultured cells and a genetically engineered mouse model that a specific palladin isoform (85–90 kDa) is strongly upregulated in tumor-associated fibroblasts of pancreatic adenocarcinomas. It is well established that when compared to quiescent fibroblasts, TAFs display characteristic changes in gene expression and cell behavior, and are more contractile, motile and secretory [25], [26]. Recently, a study using an innovative cell culture system demonstrated that tumor-associated fibroblasts may actually “lead the way” for tumor cells to invade through the basement membrane, by generating channels within the matrix that create passageways for the metastasizing tumor cells [27]. Thus, the current paradigm is that TAFs are critical players in the process of tumor metastasis, pointing to the importance of understanding the molecular mechanisms that control the acquisition of the reactive TAF phenotype.

Previously, palladin was shown to be rapidly upregulated in activated myofibroblasts that participate in wound closure in the skin [13]. Our current results show that a similar isoform-specific upregulation of palladin occurs in TAFs. It is important to note that TAFs are sometimes referred to as tumor-activated myofibroblasts because of the striking similarities in phenotype and patterns of gene expression observed between the myofibroblasts that participate in wound-healing and the activated fibroblasts that surround desmoplastic tumors. Taken together with previously published results on skin myofibroblasts, our findings support the view that upregulation of palladin may play an important role in the changes in cell morphology and behavior that accompany the acquisition of the reactive fibroblast phenotype in multiple physiological settings.

These results generate new questions regarding palladin's contribution to TAF behavior. Previous studies using a variety of cultured cell models have established that palladin plays a critical role in the formation of actin-based structures involved in cell motility and contractility, such as stress fibers, dorsal ruffles and podosomes [10], [15], [28], [29]. Thus, one likely possibility is that upregulation of 85–90 kDa palladin in TAFs promotes the motility of these cells. A second possibility is that palladin upregulation contributes to their enhanced contractility. It has been demonstrated that the increased stiffness of tumors, as compared to normal tissue, generates mechanical signals that are pro-proliferative and pro-invasive [30]. These mechanical signals originate, in part, from the enhanced contractility of the muscle-like TAFs. The 85–90 kDa palladin isoform has been shown to contribute to the contractility of vascular smooth muscle cells [9], a cell type that is phenotypically similar to TAF. In smooth muscle cells, palladin may promote contractility both directly via enhanced actin bundling and indirectly, by regulating the expression of cytoskeleton-associated proteins including myosin, actin, calponin and h-caldesmon [9]. Given the similarities in gene expression and behavior that exist between TAFs and smooth muscle cells, it will be a high priority in the future to determine if 85–90 kDa palladin contributes to enhanced contractility in TAFs. The precise mechanism by which palladin may influence the levels of other contractile proteins is not known; however, palladin has been detected in the nucleus of multiple cell types and thus may play a role in transcriptional regulation [17], [28]. In our IHC results, intense nuclear staining was observed in normal pancreatic acinar cells when tissue sections were stained with pan-palladin polyclonal antibodies. Thus, an important new direction for future projects will be to identify the palladin isoforms that enter the nucleus and explore their function in that subcellular compartment.

It is important to place our results within the context of earlier findings by multiple groups that have explored palladin expression in the context of tumorigenesis. Palladin was previously shown to contribute to the invasive motility of human breast cancer cell lines, in a study that focused exclusively on the expression of 85–90 kDa palladin [15]. In light of the current results, it is interesting to note that the most invasive breast cancer cell lines used in the previous study all display mesenchymal features, suggesting that they derive from tumor cells that have undergone epithelial to mesenchymal transition. In the future, it may be informative to revisit those human breast cancer cell lines using multiple palladin antibodies, to ask if other palladin isoforms are expressed in those cells, and if the isoforms conform to cell-type specific, epithelial versus mesenchymal, patterns of expression. In addition, a previous PCR-based study of a rare familial form of PDA showed that palladin mRNA levels are increased in pre-invasive lesions relative to normal pancreas, suggesting that palladin upregulation may be a marker for early stages in the development of this familial form of the disease [5], and our current results obtained using a mouse model of PDA support this idea. Also, our results are in good agreement with a previous analysis of palladin expression that was achieved by staining of a tumor microarray, in which palladin was found to be overexpressed in >96% of pancreatic adenocarcinomas [6]. We have extended these studies by IHC and immunoblot analyses of patient samples to show that upregulation of 85–90 kDa palladin is a consistent feature of pancreatic ductal adenocarcinomas. Thus, much of the apparent discrepancies among various previous studies of the level and localization of palladin may have resulted from the use of antibodies with different specificities for distinct palladin isoforms. In future studies, it will be important to determine if upregulation of 85–90 kDa palladin is an essential early step in the evolution of the tumor microenvironment.

The high mortality of pancreatic cancer is attributable to a lack of screening tests, inaccessibility of the pancreas, and late cancer stage at diagnosis [1]–[3]. To improve this dismal prognosis, attempts are being made in the U.S. and Europe to identify risk factors and screening methodologies. Currently endoscopic ultrasound sonography (EUS) is the most sensitive screening test available. It can identify early pancreatic changes caused by an IPMN (pre-malignant intraductal papillary mucinous neoplasm), mucinous cysts, or malignant lesions (small masses and PanIN lesions, i.e., preneoplastic pancreatic intraepithelial neoplasia), or early features of chronic pancreatitis [31]. Our current results indicate that upregulation of a 85–90 kDa palladin isoform is specific to pancreatic ductal adenocarcinoma, and is not detected in less invasive tumor types. Our results also show that palladin expression can be detected in small amounts of tissue (10–15 mg). Taken together, these results point to the possibility that immunodetection of the 85–90 kDa palladin isoform in samples collected by EUS-guided fine-needle aspiration may have diagnostic utility as an early, specific marker for the development of pancreatic adenocarcinoma, which may provide new avenues for diagnosing pancreatic cancer at a treatable stage of the disease.

## Materials and Methods

### Ethics Statement

This study was conducted according to the principles expressed in the Declaration of Helsinki. The study was approved by the Institutional Review Board of UNC Hospital (#07-2046). All patients provided written informed consent for the collection of samples and subsequent analysis. All animals were handled in strict accordance with good animal practice as defined by the relevant national and/or local animal welfare bodies, and all animal work was approved by the “Fred Hutchinson Cancer Research Center Institutional Animal Care and Use Committee” (Institutional Animal Welfare Assurance Number A3226-01).

### Materials

Four different antibodies against palladin were used: two rabbit polyclonals, 622 and commercial (ProteinTech Group) and two monoclonals, 1E6 and 4D10. The commercial antibody was utilized previously for IHC and immunoblot studies [6], polyclonal 622 is the sister antibody of the previously characterized 621 [5], and monoclonals 1E6 and 4D10 were characterized previously [10], [15]. To control for equal sample loading, antibodies to alpha tubulin (Lab Vision Corporation), GAPDH (Santa Cruz) and α-smooth muscle actin (Sigma) were used. The protease inhibitor cocktail for mammalian tissues was from Sigma. Alexafluor-488 and Alexafluor-568 anti-mouse IgG and anti-rabbit IgG conjugated secondary antibodies were from Molecular Probes.

### Cell lines

Human cell types used in this study include: pancreatic ductal epithelial cells (HPDE) [5], three pancreatic cancer cell lines (PANC1, MiaPaCa and ASPC1), primary normal gingival fibroblasts (NF), primary tumor-associated fibroblasts obtained from human pancreatic adenocarcinomas (TAF), and immortalized pancreatic stellate cells. Pancreatic cancer cell lines were obtained from ATCC: PANC1 (ATCC # CRL-1469), ASPC1 (ATCC # CRL-1682) and MiaPaCa (ATCC # CRL 1420). Primary cultures of human gingival fibroblasts were obtained from biopsies of normal gingiva in patients aged 10 to 16 years, as described previously [32], [33], and were used at passages 3–12 for all experiments. Human pancreatic tumor-associated fibroblasts were isolated as described previously [34] and used at passage 2–4. Immortalized human pancreatic stellate cells were characterized previously [20]. NF were cultured in MEM Alpha plus 15% FBS and antibiotics, while TAF and stellate cells were cultured in DMEM plus 10% FBS and antibiotics. HPDE cells were cultured in Keratinocyte SFM with 50 mg Bovine Pituitary Extract and 2.5 µg Epidermal Growth Factor. Panc1 and MiaPaCa cells were cultured in DMEM plus 10% FBS and antibiotics. ASPC1 were cultured in RPMI 1640 plus 10% FBS and antibiotics. All cultured cells were grown at 37°C and 5% carbon dioxide.

### Cell Lysis and Immunoblot Analysis

Cells were cultured on 100 mm tissue culture dishes, rinsed briefly with phosphate-buffered saline, and then scraped into a lysis buffer containing 50 mM Tris (pH 7.0), 150 mM NaCl, 1% Triton X-100, and a protease inhibitor cocktail for mammalian tissues. The supernatant was collected after centrifugation at 14,000 rpm for 15 min. The cell lysates were analyzed by immunoblot or frozen with liquid nitrogen and stored at −80°C for future use. For the immunoblot, lysates were boiled in 2X Laemmli buffer, and 20 µg of protein were resolved by SDS-PAGE in each lane of a 4–12% gel. The proteins were transferred to nitrocellulose and immunoblotted. Immunocomplexes were visualized using the Western Lights Chemiluminescence Detection kit from Perkin-Elmer.

### Immunohistochemistry

De-identified patient samples of primary and metastatic tumors were obtained through an IRB-approved study supported through the tissue procurement facility of the Lineberger Comprehensive Cancer Center. The protocol supports the procurement of malignant and non-malignant tissue for cancer-related research, and informed consent is obtained from patients who agree to participate. Five micron-thick, formalin-fixed paraffin-embedded (FFPE) tissue sections were collected on a coated glass slide, dried vertically overnight at room temperature and heated in a 60°C drying oven for one hour. The tissue sections were de-paraffinized in two changes of xylene, hydrated using descending grades of ethanol, and rinsed in a Tris buffer containing Tween 20 (DakoCytomation TRIS-Buffered Saline S1968/DakoCytomation Tween 20 S1966). Endogenous peroxidase activity was blocked by incubating in a 3% hydrogen peroxide/methanol solution for 10 minutes. (Fisher Scientific Hydrogen Peroxide ACS H325/Mallinckrodt Chemicals Methanol 3016-16). Antigen retrieval was performed by heating with steam in a citra buffer (BioGenex Antigen Retrieval Citra HK086-pK) for 30 minutes, cooling for 15 minutes at room temperature and placing in Tris buffered solution with Tween for 5 minutes. Immunostaining was performed on the Dako Autostainer platform (DakoCytomation) at room temperature. Briefly, each tissue section was incubated in normal horse serum (Vector Laboratories Elite kit 6102) for 15 minutes; the primary antibody was applied for 60 minutes, and detection was completed by incubating with a biotinylated link. An avidin-biotin complex (Vectastain Elite Kit 6102) was applied for 30 minutes followed by diaminobenzidine (Innovex NB314SB) chromogen for 2 minutes. Signal contrast was maximized by counterstaining in hematoxylin (DakoCytomation Mayer's Hematoxylin S3309) for 1 minute, rinsing in deionized water and finally in a bluing solution (Richard-Allan Scientific 7301) for 30 seconds. Samples were then rinsed in Tris buffer for 5 minutes, dehydrated in ethanol and placed in xylene, and mounted using Permount (Fisher Scientific Permount SP15).

### Immunoblot of patient samples

Pea-sized pieces of fresh tissue were snap-frozen in liquid nitrogen, ground in a chilled mortar and pestle, and extracted in lysis buffer containing 50 mM Tris pH 7.5, 8 M urea, 5% SDS, 10 mM EDTA, and protease inhibitor cocktail for mammalian tissues. Samples were then centrifuged at 15,000×g to remove any unsolubilized particulates, and the supernatant was resolved by SDS-PAGE, using 15 µg protein loaded per lane.

### Mouse Tumors

Primary pancreatic ductal adenocarcinomas and metastases were isolated from genetically engineered mice in which an activating mutation in *Kras* is targeted to progenitor cells of the developing pancreas [21]. These animals develop pre-invasive ductal lesions stochastically and manifest the full spectrum of PanIN lesions culminating in invasive and metastatic disease with clinical, histopathological and molecular features that faithfully mimic the human disease [21], [22], [23]. Tissue specimens were dissected, formalin-fixed and paraffin-embedded and sectioned for immunohistochemistry, as described above for the human samples.

### RNA isolation and quantitative reverse transcription-PCR

De-identified patient samples were collected after approval by each individual IRB and use of all samples for this study was approved by the UNC IRB. Samples of matched normal pancreas and pancreatic cancer were obtained at the time of operation and flash frozen in liquid nitrogen. Total RNA was extracted from the snap-frozen tumor samples using Allprep Kits (Qiagen) and quantified using nanodrop spectrophotometry (ThermoScientific). Reverse transcriptase reactions were performed using the commercial kit High-Capacity cDNA Reverse Transcription (Applied Biosystem) and contained up to 2 µg of total RNA. Real-time quantitative PCR was performed using an Applied Biosystems 7500 Fast Real Time PCR System in a a total volume of 25 µl mixture containing 2.5 µl of 10-fold diluted cDNA, 0.2 pM of sense and antisense primers and the Power SYBR@ Green Master Mix (Applied Biosystem). Because the coding sequence of the 85–90 kDa isoform of palladin is contained and/or overlaps with the coding sequence of other palladin isoforms, RT-PCR primers were designed to amplify mRNA of the Ig3 domain containing isoforms (isoforms #1, #2, #3, #4, #5 and #7). The primers used in the experiment were as follows: sense 5′ – AAC CGA GCA GGA CAG AAC- 3′ and antisense 5′- TGG TGG CAC TCC CAA TAC-3′. The thermal cycling conditions were 50°C for 2 min, then 95°C for 10 min followed by 40 cycles of initial denaturation step of 95°C/15 s and 60°C/60 s. No-template reactions were included as negative controls in every plate. Sequence Detection Software (Applied Biosystems, Foster City, USA) results were imported into Microsoft Excel for further analysis. Raw expression levels were calculated from the average of the triplicate ddCT (RQ) values. Standard curve obtained for the primer and PCR product from a pool of samples was used as template. Each PCR reaction was carried out in triplicates.

## Supporting Information

Figure S1 Immunoblot analysis of pancreatic tumor-derived cell lines using palladin 1E6 Ab. Normal fibroblasts were used as a positive control. Pancreatic cells: normal human pancreatic ductal epithelial cells (HPDE) and three tumor cell lines: PANC1, MiaPaCA, and ASPC1. Whole cell lysates were analyzed by western blot using the monoclonal antibody 1E6. Blots were subjected to long exposure times and also stained for tubulin as a control for equal loading.
(0.24 MB TIF)

Figure S2 Immunohistochemistry of paraffin-embedded patient specimens. IHC staining was performed using standard antigen-retrieval protocols, and counter-stained with hematoxylin. Tissue sections were stained for palladin using two palladin antibodies: polyclonal COM from ProteinTech group, and monoclonal 4d10.
(8.42 MB TIF)

Figure S3 Control staining for IHC of paraffin-embedded human and mouse pancreatic tissues. IHC staining was performed as in Figure 2, except that normal rabbit serum was substituted for the primary antibody.
(10.34 MB TIF)
