# Supplementary figures and images for: Isoform-Specific Upregulation of Palladin in Human and Murine Pancreas Tumors
Source: PLoS One. 2010 Apr 26;5(4):e10347. doi: 10.1371/journal.pone.0010347 (PMC2859948; doi:10.1371/journal.pone.0010347)

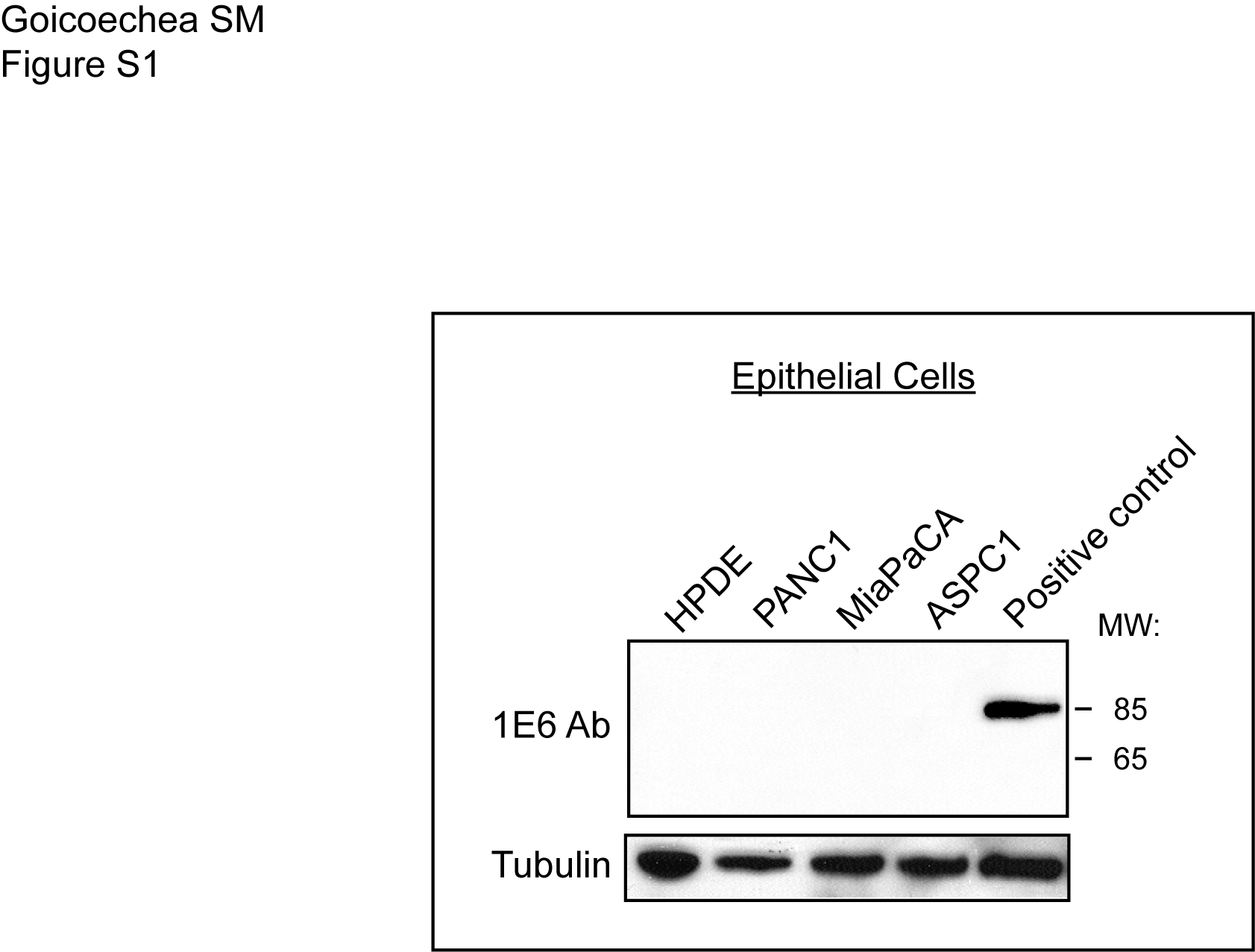

Supplement: Figure S1 — Immunoblot analysis of pancreatic tumor-derived cell lines using palladin 1E6 Ab. Normal fibroblasts were used as a positive control. Pancreatic cells: normal human pancreatic ductal epithelial cells (HPDE) and three tumor cell lines: PANC1, MiaPaCA, and ASPC1. Whole cell lysates were analyzed by western blot using the monoclonal antibody 1E6. Blots were subjected to long exposure times and also stained for tubulin as a control for equal loading. (0.24 MB TIF) [file pone.0010347.s001.tif]

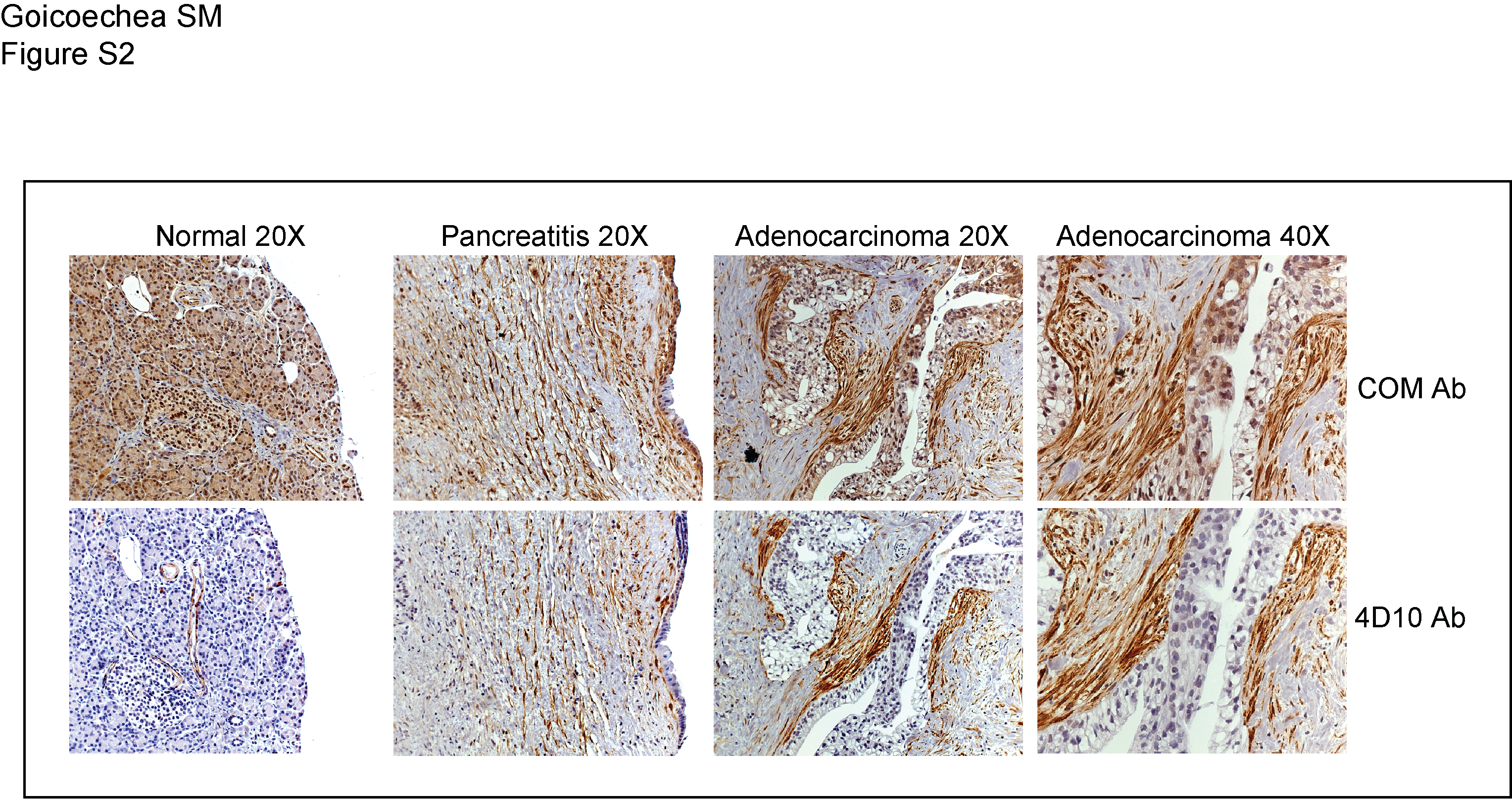

Supplement: Figure S2 — Immunohistochemistry of paraffin-embedded patient specimens. IHC staining was performed using standard antigen-retrieval protocols, and counter-stained with hematoxylin. Tissue sections were stained for palladin using two palladin antibodies: polyclonal COM from ProteinTech group, and monoclonal 4d10. (8.42 MB TIF) [file pone.0010347.s002.tif]

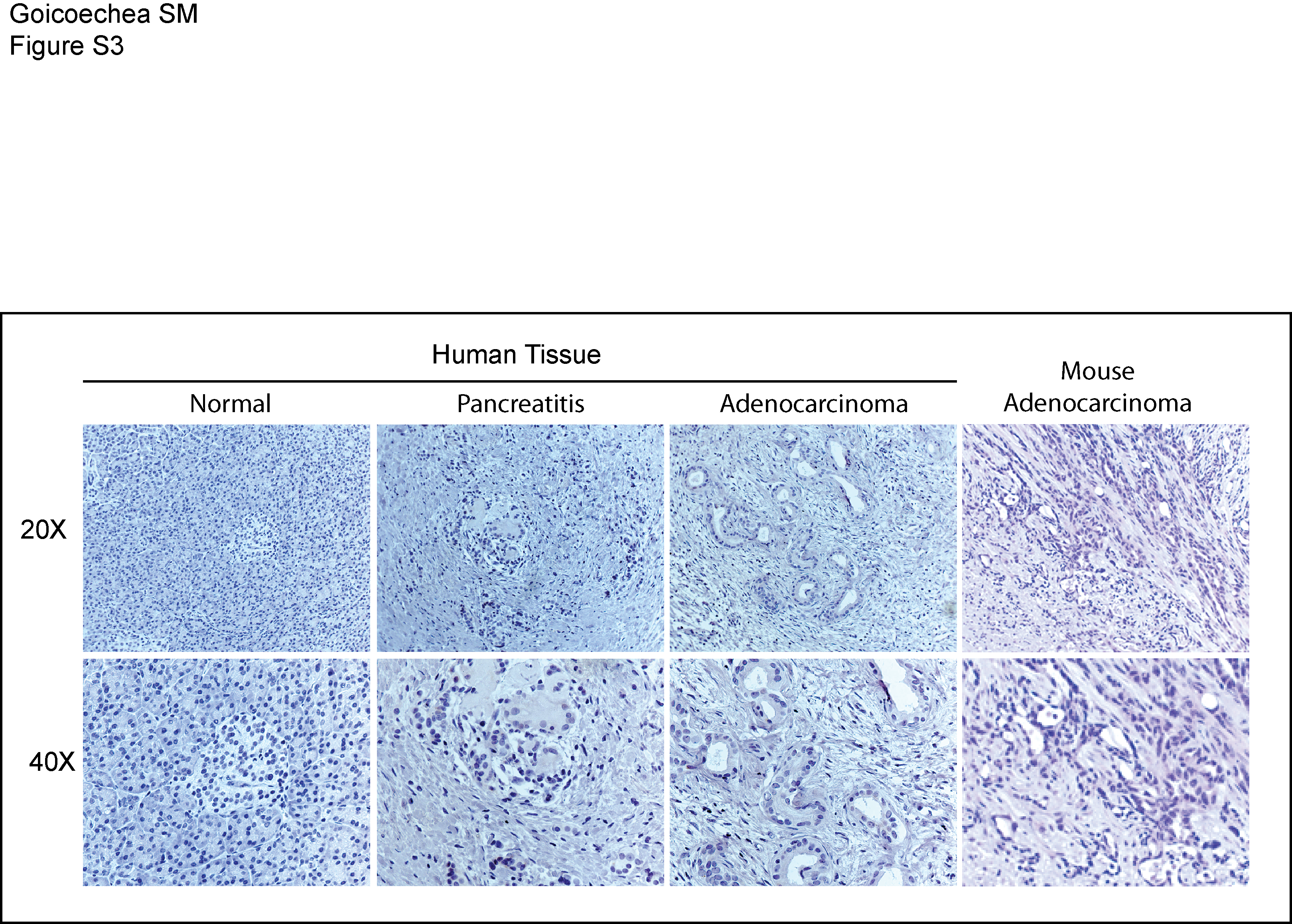

Supplement: Figure S3 — Control staining for IHC of paraffin-embedded human and mouse pancreatic tissues. IHC staining was performed as in Figure 2, except that normal rabbit serum was substituted for the primary antibody. (10.34 MB TIF) [file pone.0010347.s003.tif]
